# Esophageal erosion by a PDA occluder device following MAPC closure in tetralogy of Fallot

**DOI:** 10.1093/omcr/omag002

**Published:** 2026-02-18

**Authors:** Munaf J Yaseen, Wassan N Mohammed, Nabeeha N Akram, Mohanad K Shukur Al-Ghanimi

**Affiliations:** Department of Pediatrics, College of Medicine, Baghdad University, Baghdad, Medical City Complex, Bab al-Muadham, 61023, 12114, Iraq; Department of Obstetrics and Gynecology, College of Medicine, Mustansiriyah University, Baghdad, Yarmuk/Jinub Street area, Baghdad, 14132, Iraq; Department of Pediatrics, College of Medicine, Mustansiriyah University, Yarmuk/Jinub Street area, Baghdad Governorate, Baghdad, 14132, Iraq; Department of Pediatrics, College of Medicine, University of Babylon, Hay al-Jami’a / Adli Street, Hay al-Iskan, Al-Hillah, Babylon Governorate, Babylon, 51001, 4, Iraq

**Keywords:** tetralogy of Fallot, PDA occluder device, oesophageal erosion

## Abstract

Tetralogy of Fallot (TOF) is the commonest cyanotic congenital heart disease encountered in paediatrics. Major aortopulmonary collaterals (MAPCAs) often complicate TOF when surgical correction is delayed, and a transcatheter closure is commonly done prior to definitive surgery. Embolization of occluding devices is a known complication that typically occurs in the vessels or in cardiac chambers. We report a young boy who presented with 1-month dysphagia post-transcatheter closure of a large MAPCAs. Barium revealed a filling defect confirmed by endoscopy as an impacted occluding device in the oesophagus. It was surgically removed, and the boy was discharged well. This case highlights a rare but highly significant unreported complication.

## Introduction

Major aortopulmonary collaterals (MAPCAs) often complicate Tetralogy of Fallot (TOF), which is one of the most common congenital cyanotic heart diseases that affects the paediatric age group [1]. MAPCAs tend to rise among cases when surgical repair is delayed. Aiming to reduce shunt and to improve postoperative complications, these MAPCs are usually closed transcatheter via coil or ductal occluder, which is considered the gold standard method [1]. Although coils can be effective in small PDAs, they are associated with a higher risk of embolization, particularly in moderate to large ducts. This limitation has led to the increased use of Amplatzer Duct Occluder (ADO) devices including: Amplatzer ductal occluder type 1(ADO1) and Amplatzer ductal occluder type 2 (ADO2), which provide more stable positioning and lower rates of residual shunting or device migration. Nevertheless, these occluders still carry risk of embolization into the pulmonary arteries, and the aorta [2]. Extracardiac erosion of these devices, particularly to the oesophagus, is exceptionally rare; we present a rare case of oesophageal erosion by a PDA occluder following a MAPCAs closure that was successfully retrieved by surgery with a favourable outcome.

## Case report

**Figure 1:**
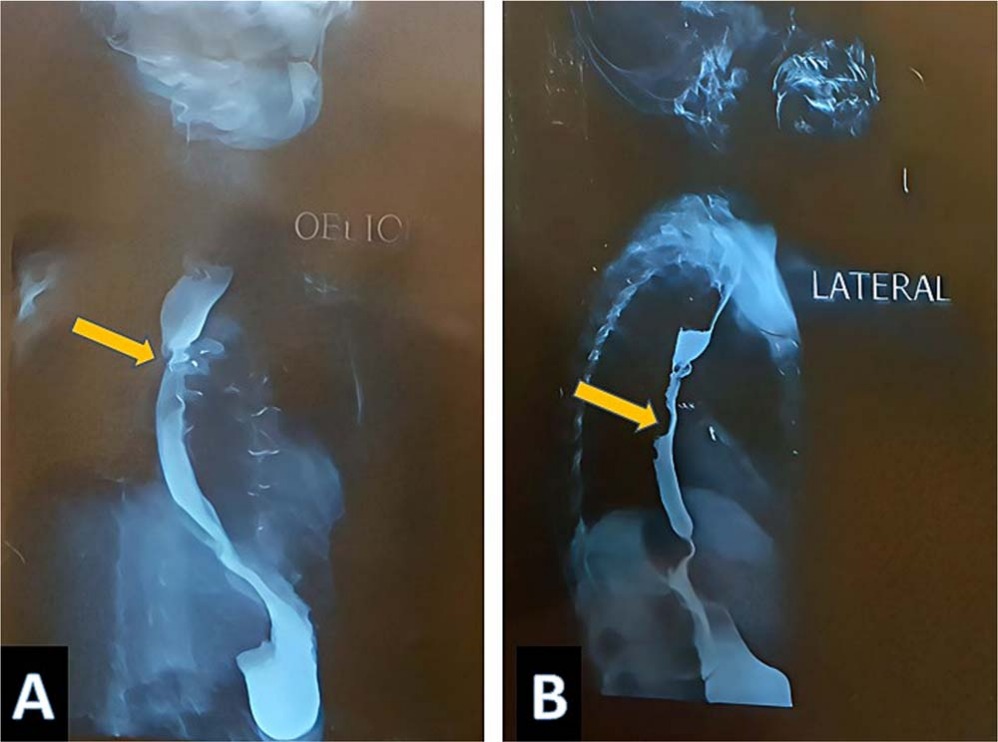
Barium meal shows a filling defect marked by yellow arrow.

**Figure 2:**
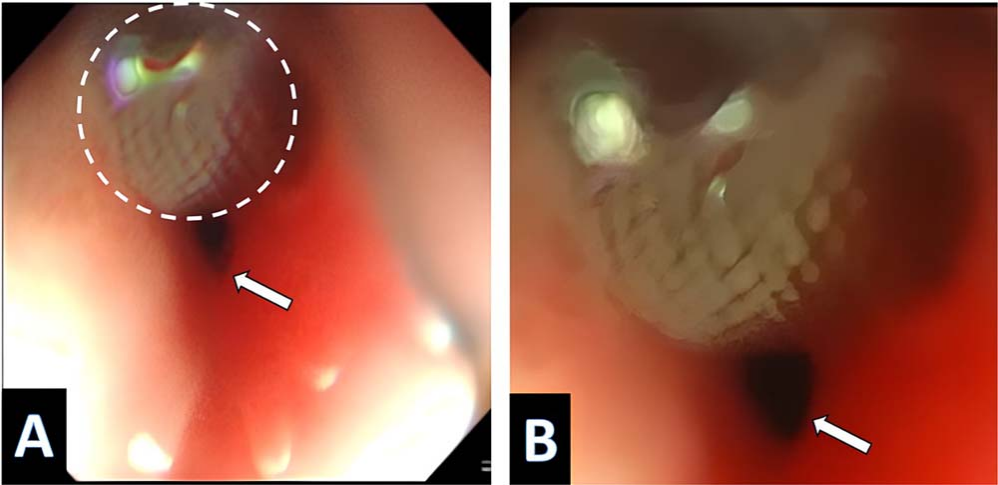
The endoscope picture shows the PDA device inside the oesophagus. Magnification by X20; (B) magnification by X40.

A three-year-old Iraqi boy with TOF diagnosed since birth, residing between Iraq and Turkey, was prepared for total surgical correction. As a preparatory step, a transcatheter study was conducted in Istanbul. The results showed three significant MAPCAs, two of them were embolized with 7 × 5 mm coils, while the largest one was occluded using an 8–6 mm PDA device in a smooth, uneventful surgery. One month after the surgery, the boy presented in Baghdad with progressive dysphagia for solid food with weight reduction from 11 kg (on the 5^th^ centile for age) to 10.2 kg (below the 5^th^ centile for age). Physical examination and lab tests were unremarkable, which led to a gastroesophageal regurgitation as a possible underlying cause. Imaging study: Bairam Meal revealed a mid-oesophageal filling with no mediastinum leakage, without suggestion of a filling defect nature, (Fig. 1A and B). Endoscopic examination of the oesophagus shows a PDA device protruding and impacting the upper part of the oesophagus, (Fig. 2A and B). This implies that the PDA occluder had eroded the collateral vessels into the oesophagus lumen to manifest as dysphagia. The case was referred to a cardiothoracic surgeon, where surgical retrieval was performed by open thoracotomy. The device was removed successfully without injury to the surrounding structures, the oesophagus was sutured but without residual defect and no stent was placed, and the postoperative course was uneventful.

The operation and postoperative period went smoothly without complications, and the boy was discharged home three days later. There was complete resolution of dysphagia at follow-up visits with 1-week.

## Discussion

All cyanotic congenital heart diseases, including TOF, may develop MAPCAs when surgical repair is delayed. The MAPCAs proliferate and inversely impact pulmonary vascular development, uneven pulmonary perfusion, and increase right ventricular workload, thus imposing long-term outcomes [1, 3]. Selective Transcatheter closure of these abnormal vessels can decrease the hemodynamic burden on the heart and improve preoperative haemodynamic and postoperative recovery after total surgical correction. It is worth noting that this patient’s procedure was performed at a different institution to where the complication was identified; at our institution practice consists of choosing the smallest effective closure device, placing the device as distally as possible to avoid erosion risks to adjacent structures (such as the oesophagus) and never using a device larger than the ADO1 5-4 mm. [4].

These vascular occluders do have complications such as vascular injury, erosion, and migration. Migration of PDA occluder device is known complication, it can be early within hours- days (1%–3%) or late (<0.03%) years after their placement [5, 6]. Common sites for device migration reported in the literature are the descending thoracic aorta and the pulmonary vasculature [4]. However, device migration to oesophagus is very rarely reported and usually presented with upper gastrointestinal bleeding or dysphagia. Furthermore, the diagnosis is confirmed by Echo, fluoroscopy, or CT scan when the patient presents with unexplained decompensation or compromise [7]. The retrieval is either by percutaneous snare by Cath-based technique, or it is done by open surgery when the former method fails, with a good outcome even if device erosion was reported. We have summarized some of the reported cases in the last 5-years and the outcome.

The current case is unique in more than one aspect; device migration in TOF cases is rarely reported, and most of them occur during placement. The reported cases mention that the aorta and pulmonary vessels are the most common sites. To the best of our knowledge, there are only two previous reports of oesophageal erosion by PDA occluder after MAPCAs closure, which expands the spectrum of device-related complications in CHD, (Table 1).

**Table 1:** Summary of reported cases of occluders migration to the oesophagus with related details and outcome.

Authors Year	Age, sex	Type of occluder	Underlying Defect	Presenting symptom	Retrieval	Outcome/complication
Wiegand G et al. (2016) [8]	4.5 months, Female	Amplatzer vascular plug 4 occluder	AP shunt	severe gastrointestinal bleeding	Right lateral thoracotomy, the occluder was extracted surgically, the oesophageal injury sutured.	Uneventful, none
González et al. (2020) [9]	16 years male	ADO	scimitar syndrome with 2 large AP collaterals	Progressive dysphasia	endoscopic removal of the device with stent placement in the oesophagus	The esophageal stent was removed at 3 weeks, with no residual esophageal stenosis

There are many key points from this case. Device oversizing or proximal placing can cause migration and erosion to the adjacent mediastinal structure. The anatomical proximity of the oesophagus should be considered as a migration site, even though it has not previously been reported. The importance of close follow-up and clinical vigilance to CHD cases cannot be overestimated, and the appearance of unusual symptoms (such as dysphagia) should prompt investigation. Finally, imaging studies and endoscopy are important tools in unveiling the underlying cause and device migration site.

## Notes

The parents of the patient gave signed informed consent for publication of the case with all related pictures and information.

**Data access:** The data that supports the findings of this report available from the corresponding author upon reasonable individual request.

The paper has not been previously published, nor is it concurrently submitted to any other journal, The final version of the manuscript has been read and approved by all authors.
